# Formation of Nanodiamonds during Pyrolysis of Butanosolv Lignin

**DOI:** 10.1021/acsnano.4c02950

**Published:** 2024-08-23

**Authors:** Yi Feng, Daniel J. Davidson, Weihao Sun, Valentina Milani, Grant W. Howieson, Nicholas J. Westwood, Wuzong Zhou

**Affiliations:** † EaStChem, School of Chemistry, University of St Andrews, Fife, St Andrews KY16 9ST, U.K.; ‡ Biomedical Sciences Research Complex, University of St Andrews, North Haugh, Fife, St Andrews KY16 9ST, U.K.

**Keywords:** nanodiamond, lignin, pyrolysis, multiple nucleation, electron microscopy

## Abstract

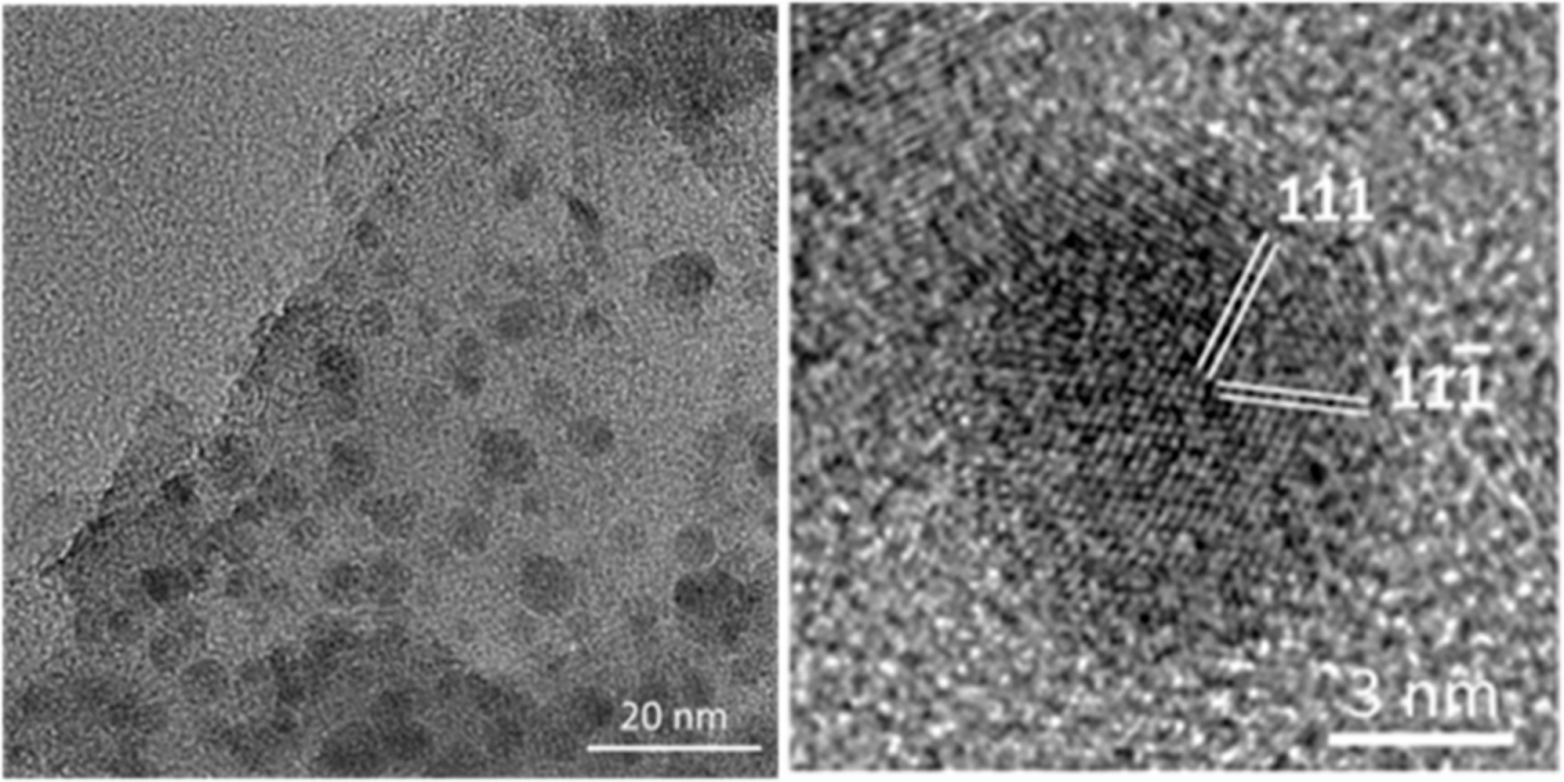

The preparation of artificial diamonds has a long history driven by decreased costs compared to naturally occurring diamonds and ethical issues. However, fabrication of diamonds in the laboratory from readily available biomass has not been extensively investigated. This work demonstrates a convenient method for producing nanodiamonds from biopolymer lignin at ambient pressure. Lignin was extracted from Douglas Fir sawdust using a butanosolv pretreatment and was pyrolyzed in N_2_ at 1000 °C, followed by a second thermal treatment in 5% H_2_/Ar at 1050 °C, both at ambient pressure. This led to the formation of nanodiamonds embedded in an amorphous carbon substrate. The changes occurring at various stages of the pyrolysis process were monitored by scanning electron microscopy, Fourier transform infrared spectroscopy, and nuclear magnetic resonance spectroscopy. High resolution transmission electron microscopy revealed that nanodiamond crystallites, 4 nm in diameter on average, formed via multiple nucleation events in some carbon-containing high density spheres. It is proposed that highly defected graphene-like flakes form during the pyrolysis of lignin as an intermediate phase. These flakes are more deformable with more localized π electrons in comparison with graphene and join together face-to-face in different manners to form cubic or hexagonal nanodiamonds. This proposed mechanism for the formation of nanodiamonds is relevant to the future fabrication of diamonds from biomass under relatively mild conditions.

Carbon materials have attracted increasing scientific attention because of their properties in a wide range of applications, including CO_2_ capture, catalysis, gas storage, electrode materials, etc.^1,2^ One of the main sources of these materials is biomass, so the development of carbon materials from biomass is now an important research area in green chemistry. The commonly synthesized bulk carbon forms are amorphous carbon and graphite. However, diamond is rarely mentioned because its preparation requires extreme conditions, i.e., very high temperature and pressure.^3^

Nanoscale carbon materials are even more interesting. The most commonly investigated nanocarbons are one-dimensional (1D) carbon nanotubes and two-dimensional (2D) graphene.^4,5^ Zero-dimensional carbon materials, including fullerenes and carbon quantum dots, often show interesting chemical properties. For example, graphite-type carbon dots supported on carbon nitride films show excellent catalytic activity in the selective reduction of CO_2_ to methanol using pure water.^6^

Nanodiamonds are also scientifically important materials and have seen in use in a variety of applications due to their interesting properties.^7^ They are inherently biologically compatible and have demonstrable biomedical applications such as in enhanced tissue repair^8^ and drug delivery systems.^9^ The optical properties of nanodiamonds (and other nanocarbons such as carbon quantum dots) have seen them used as a substrate to design materials for optoelectronic applications.^10^ Mixed carbon materials containing nanodiamonds embedded in graphite have been used in lubricant oils to enhance fuel efficiency in diesel engines with what is proposed to be a combined friction-reducing and polishing effect.^11^ They can be produced in explosions or meteoritic impacts.^12^ In fact, detonation synthesis of nanodiamonds is the industry standard for their commercial production.^13^ In this process, high pressure and high temperature are crucial conditions. Other methods for the synthesis of nanodiamonds have also been developed,^14^ e.g., hydrothermal synthesis,^15^ chemical vapor deposition,^16^ laser bombardment,^17^ etc. Most of the synthetic processes that lead to nanodiamonds require harsh conditions. The hydrothermal synthesis drew our particular attention, as in this process nanodiamonds can be produced from polycyclic aromatics at relatively low pressure and temperature. This approach was supported by lattice energy calculations, indicating that when the surface is terminated with hydrogen, nanodiamonds smaller than 3 nm in diameter could be more stable than graphite or aromatic polycyclic precursors.^18^

In recent years, the formation of nanodiamonds from biomass has been investigated. The use of both lignin and cellulose has drawn people’s attention due to their very high abundancy on the Earth. Lin et al. used high energy lasers with nanolignin/cellulose nanofibril composite films to generate locally high temperatures and pressures. After laser writing, some nanodiamonds were detected.^19,20^ This approach demonstrated that lignin is an important resource for producing nanodiamonds. Unfortunately, the products from laser writing contain other crystalline carbon forms, such as graphene and onion-like carbon. Furthermore, the large-scale production of nanodiamonds using this method would be difficult. Although a formation mechanism of nanodiamonds from lignin was discussed by these authors, a continued discussion of the proposed phase transformations is of importance, as, for example, additional details of the conversion of onion-like carbon to nanodiamond could be developed. More generally, any additional insight into the mechanism of conversion of biomass to nanodiamonds is important, as this area of research develops.

A typical type of nanodiamond material has a face-centered cubic structure with a unit cell parameter of *a* = 3.567 Å. Another rarer type of diamond exists and has a hexagonal unit cell with parameters of *a* = 2.52 and *c* = 4.12 Å.^21^ The latter is known as lonsdaleite. While the synthesis of diamonds from graphite is known to require extreme conditions, nanodiamonds have been detected in a candle flame.^22^ This implies that at a relatively low temperature and ambient pressure, burning organic wax molecules can form cubic nanodiamonds if suitable precursor molecules can be generated during combustion. Other types of carbon nanoparticles, such as fullerenes and graphitic spheres, were also detected in the candle flame.^22^ Producing nanodiamonds and other carbon nanoparticles by combustion has little value as a practical approach, as the vast majority of intermediate particles will continue to burn, giving CO_2_ rather than potentially expanding the size of the nanodiamonds. A synthetic pathway to nanodiamonds using cost-effective raw biomass materials, an environmentally friendly process and mild conditions that leads to a stable form of products, remains highly desirable and needs to be developed.

We report here the extraction of high quality lignin from Douglas Fir wood using a butanosolv pretreatment^23^ and, via pyrolysis of this lignin, the formation of nanodiamonds embedded in an amorphous carbon substrate. The step-by-step process of pyrolysis is discussed, supported by the results of Fourier transform infrared (FTIR) and nuclear magnetic resonance (NMR) studies. Structural characterization of nanodiamonds was performed by using high resolution transmission electron microscopy (HRTEM). A formation mechanism of nanodiamonds is proposed with the most important step being a face-to-face connection of 2D highly defected graphene-like lignin flakes. Lignin accounts for approximately 30% of the weight of wood and is therefore one of the most abundant biomass-sourced raw materials in the world.^24^ This invented method will inspire future research aimed at turning biomass into useful carbon materials by using a convenient pathway.

## Results and Discussion

**Figure 1:**
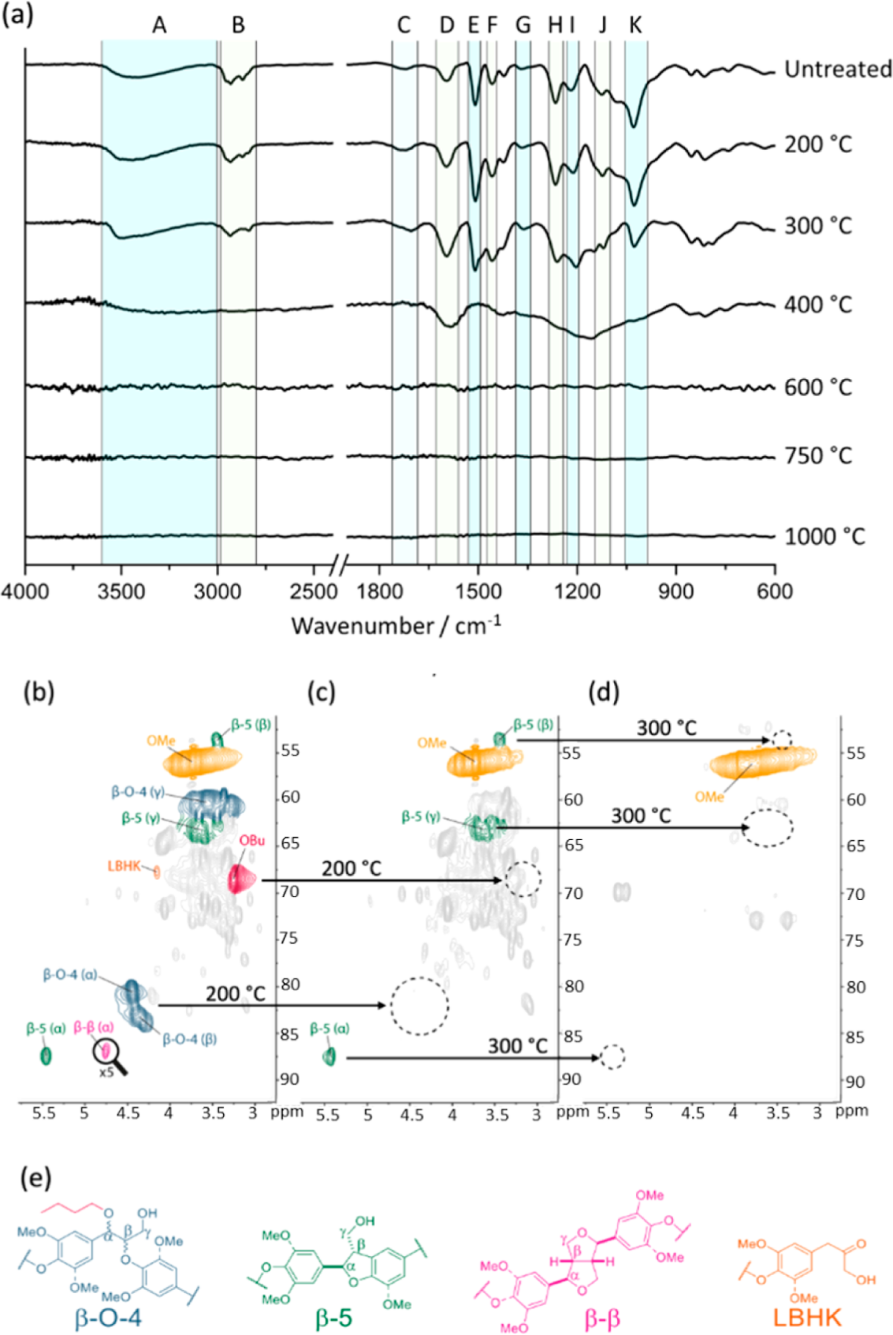
(a) FT-IR spectra of butanosolv lignin and residue after pyrolysis at various temperatures. Regions of absorption frequencies corresponding to relevant structural features in lignin based on literature assignments^28−35^ are highlighted: A—3600–3000 cm^–1^, O–H stretch; B—2950–2850 cm^–1^, aromatic and alkyl C–H stretch; C—1730 cm^–1^, C=O stretch in oxidized units; D—1600 cm^–1^, aromatic C=C stretch; E—1510 cm^–1^, aromatic skeletal vibrations associated with C–H deformations; F—1460 cm^–1^, aromatic C–H deformations; G—1360 cm^–1^, aliphatic C–H bend (not in OMe); H—1260 cm^–1^, guaiacyl breathing vibrations; I—1220 cm^–1^, aromatic C–O stretch; J—1120 cm^–1^, aromatic C–H in-plane deformation; K—1030 cm^–1^, aliphatic C–O deformation. HSQC NMR (700 MHz, DMSO-*d*_6_) spectra of the linkage region of (b) butanosolv lignin before pyrolysis; (c) DMSO-soluble component of residue after pyrolysis at 200 °C; and (d) DMSO-soluble component of residue after pyrolysis at 300 °C. (e) Structures of relevant lignin interunit linkages that correspond to signals shown in HSQC NMR spectra. The cross peak signal corresponding to the β–β Hα-C position is shown in (b) with the threshold decreased five times due to low abundance. Structural features that change upon increasing pyrolysis temperature are highlighted by dashed black circles in the HSQC NMR spectra.

Lignin is typically isolated from wood samples using a pretreatment. In this case, a butanosolv (BoS) pretreatment process^25^ was used to prepare a lignin from commercially available Douglas Fir sawdust (see Methods section for details). Douglas Fir wood was selected as it is a softwood, and therefore the lignin is relatively simple, with the dominant aromatic subunit within the lignin being a guaiacyl (G) unit. Previous work by Rowlandson et al.^26^ has demonstrated that successful carbon material formation from a range of ethanosolv lignins is dependent on the nature of the dominant unit in the starting lignin. However, a recent modification of their protocol decreased variability in the structure of the carbon material produced from different ethanosolv lignins.^27^ Our obtained lignin (isolated in 10 wt % yield) was characterized by FTIR, solution-state 2D heteronuclear single quantum coherence (HSQC) NMR, and scanning electron microscopy (SEM) (Figures 1 and S1). As expected,^23^ the starting lignin was globular in structure and of good quality with a high β-O-4 linkage unit content, of which the majority was present in the α-butoxylated form. NMR signals consistent with the presence of β-5 and β–β units were also present in the starting lignin.

FTIR analysis of the untreated starting butanosolv lignin showed the presence of the characteristic absorption bands observed for lignin,^28−33^ including (i) O–H groups (Figure 1a, region A, 3600–3000 cm^–1^); (ii) C–H bonds (region B, 2950–2850 cm^–1^); (iii) C=O groups (region C, 1730 cm^–1^); (iv) C=C bonds due to the presence of aromatic rings (region D, 1600 cm^–1^); and (v) C–O groups (regions I and K, 1220 and 1030 cm^–1^). The lignin was then heated in a tube furnace under a nitrogen atmosphere to different temperatures to investigate the early stages of pyrolysis (see Methods section for details). An overall decrease in the intensity of the bands (relative to the other signals) between 2950 and 2850 cm^–1^ (C–H stretches, region B, Figure 1a) and 1030 cm^–1^ (aliphatic C–O deformations, region K) on partial pyrolysis of the lignin at 200 °C was assigned to the loss of aliphatic ether functional units within the lignin. This was supported by the NMR analysis of the deuterated dimethyl sulfoxide (DMSO-*d*_6_)-soluble component of the partially pyrolyzed sample (c.f. Figure 1b,c,e, loss of signals corresponding to the β-O-4 units and the butanol component). The β-5 unit was clearly still present in the soluble product from the 200 °C treated lignin, but more detailed inspection of the NMR data showed that the lignin-bound Hibbert ketones, and the majority of the β–β units had not survived.

Heating of the lignin at 300 °C led to a further change in the structure of the DMSO-soluble material, which now contained no recognizable lignin interunit linkages in the 2D HSQC NMR analysis (Figure 1d,e). This stepwise degradation of the starting lignin as a function of (partial) pyrolysis temperature has not been studied by solution-state NMR previously to the best of our knowledge and therefore provides additional insights into the early stage of the lignin to carbon transformation. When a pyrolysis temperature of 300 °C was used, evidence to support the increased presence of C=C bonds (likely in aromatic rings) was obtained from the FTIR analysis (Figure 1a, see changes in relative intensities of peaks at 1600 cm^–1^, region D). The increase in the presence of aromatic rings during the thermal treatment was also supported by NMR analysis (Figure S1a–c). Significant structural changes also occur between 300 and 400 °C where Stage I pyrolysis ends and Stage II pyrolysis begins.^29,30^ These changes were consistent with the loss of the methyl in methoxy functionalities, which results in increased phenolic and catechol content, aromatic C–O–C bonding between different aromatic rings, and increased relative C–C and C=C bonding character. These observations, leading to a polycyclic aromatic hydrocarbon structure (see Figure 6c below), were consistent with literature reports^31−33^ but had not been previously observed for butanosolv lignins.

When the pyrolysis was carried out at 600 or 1000 °C, no DMSO-soluble material was obtained, consistent with no remaining “lignin-like” material. FTIR analysis of the pyrolysis product from these higher temperature experiments clearly indicated the absence of the signals previously assigned to the lignin. The flat-lined FTIR spectra (Figure 1a) were taken as evidence that full pyrolysis of the biomass to carbon had occurred without any FTIR detectable additional functional groups remaining. These results were in agreement with previous reports on the thermal decomposition of lignin^36^ and the thermal gravimetric analysis (TGA) results (Figure S2), which indicated the majority of the structural changes and loss of oxygen containing functionalities was complete by 600 °C. Studies next turned to the detailed structural analysis of the samples after full pyrolysis.

The final carbon specimen formed by pyrolysis of the lignin has a very rough surface (Figure S3a). More details of the particle morphology were revealed in the SEM images. Typical SEM images (Figures S1e and S3b) show a shard-like, macroporous structure of the carbon material that contrasts with the appearance of the starting lignin (Figure S1d). Some large cages, 100 to 400 μm in diameter, with thin walls are visible. Energy-dispersive X-ray spectroscopy (EDX) confirms that the final sample contains almost exclusively carbon (Figure S3c), although the existence of hydrogen cannot be ruled out. The EDX elemental mapping also shows an even distribution of carbon across wide areas of the sample (Figure S3d). The powder X-ray diffraction (XRD) pattern of the carbon sample shows that the major component of the sample is amorphous. Some very weak diffraction peaks are also observed. Most of them cannot be indexed to any known crystalline phases of carbon. Only two peaks at 41.4 and 72.4° of 2θ, corresponding to the *d*-spacings of 2.18 and 1.30 Å, may be indexed to (100) and (110) of the hexagonal diamond structure (Figure S3e).

**Figure 2:**
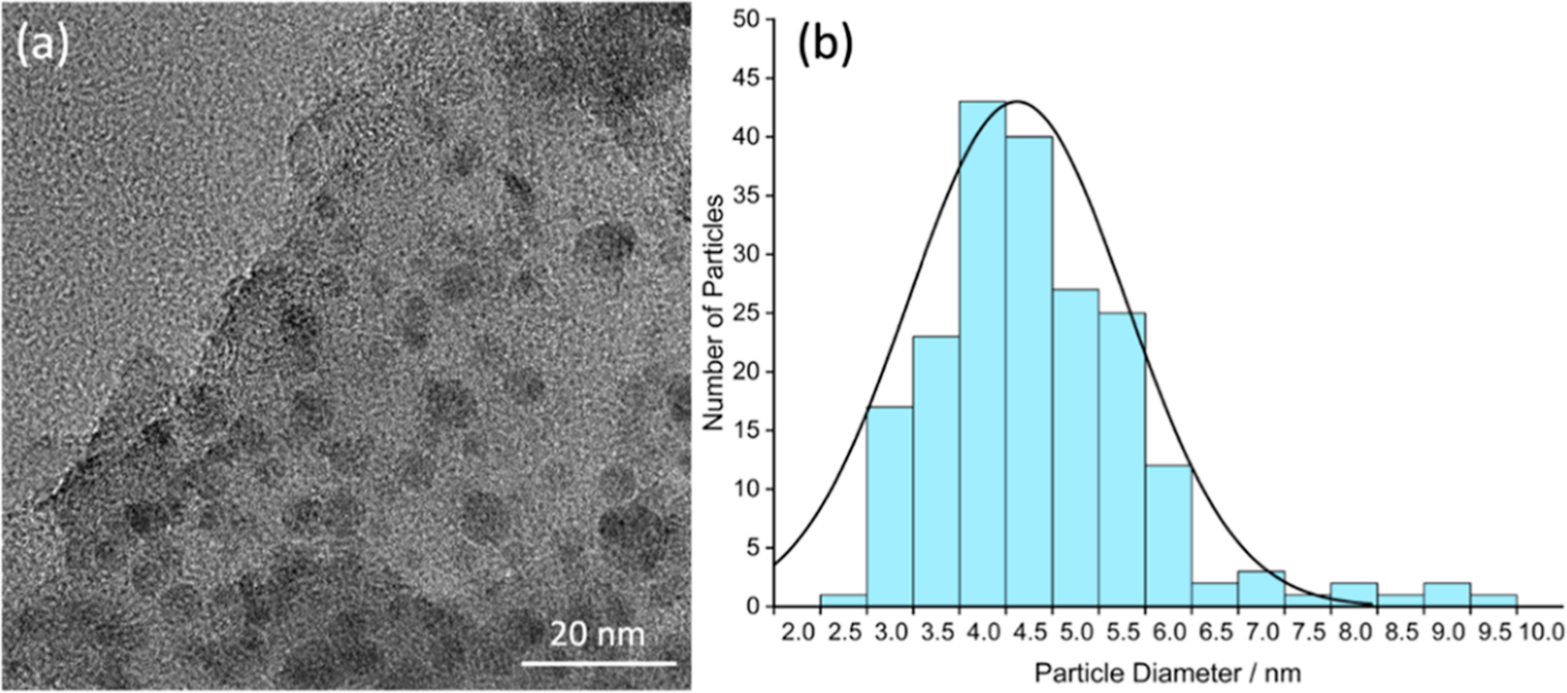
(a) Typical TEM image of carbon specimen showing embedded nanocrystallites. (b) Size distribution of nanocrystallites based on measurement of ∼200 nanocrystallites in HRTEM images.

Microstructures of the carbon specimen were investigated by using transmission electron microscopy (TEM) and HRTEM. A typical TEM image of fine particles from a ground carbon specimen (Figure 2a) shows many obvious nanoparticles embedded in the sample. As the samples are likely almost pure carbon as confirmed by EDX (Figure S3c), the dark contrast of these nanoparticles implies that their density is higher than that of the surrounding amorphous carbon according to the mass–thickness contrast formation mechanism. In fact, analysis of the high magnification HRTEM images showed that many of these nanoparticles present crystalline structures (see below for a more detailed discussion). The size distribution of these nanocrystallites (Figure 2b) was measured based on ∼200 randomly selected nanoparticles in HRTEM images (the dark dots in TEM images may not be single crystalline particles, but most likely clusters of multiple crystallites). The measured average diameter of the nanocrystallites is smaller than 5 nm. Furthermore, the volume fraction of these nanocrystallites is relatively low within the sample. It is therefore not surprising that they were not detected by powder XRD analysis (Figure S3e). On the other hand, almost all of the recorded TEM images contain many nanoparticles, indicating that the yield of nanodiamonds is not very low, although calculation of an accurate yield based on the TEM images is not possible in practice.

**Figure 3:**
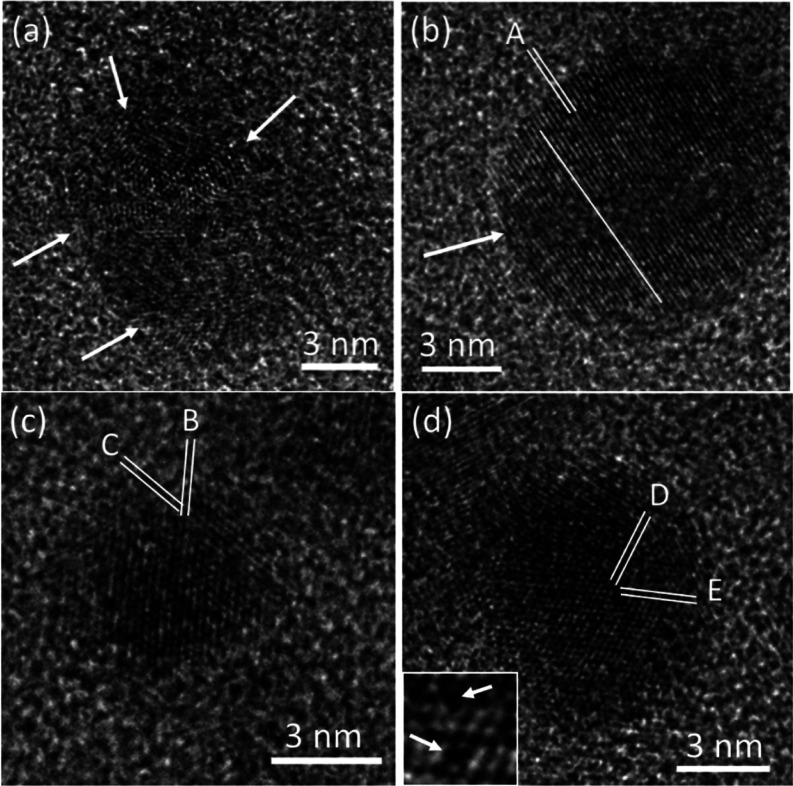
HRTEM images of typical individual nanoparticles embedded in amorphous carbon in the produced carbon material after a two stage heat treatment initially at 1000 °C in a N_2_ gas atmosphere and then at 1050 °C in a 5% H_2_/Ar mixed gas atmosphere. (a) Example of a cluster of very small crystallites. The arrows point to individual crystallites. (b) Spherical particle showing two single crystalline hemispherical sections with an amorphous region between them. Label A indicates the *d*-spacing of the fringes marked by the double line with *d*_A_ = 2.10 Å. The single line shows that the fringes in the two parts have an antiphase relationship. (c) Cubic diamond particle with *d*-spacings of *d*_B_ = 2.06 and *d*_C_ = 1.79 Å and an interplanar angle of 55°. (d) Another cubic diamond particle with *d*-spacings of *d*_D_ = *d*_E_ = 2.05 Å and an interplanar angle of 70°. The inset is an enlarged image of the central area of the crystallite, and the arrows point to dark spots with local lattice distortion.

HRTEM images were used to study the crystallinity and crystal phases of the nanoparticles. Many relatively large particles were actually clusters of very small crystallites and had a low crystallinity. Figure 3a shows one of these clusters. It seems that multiple nucleation events occurred in this particle. A large number of defects and lattice distortions can be observed, and the crystallites (1 to 2 nm in diameter) are not orientated. Figure 3b shows another particle with a more regular spherical shape. The middle region (indicated by the arrow) is still amorphous and is sandwiched by two hemispherical single crystalline parts. The measured *d*-spacing of the 1D fringes, *d*_A_ = 2.10 Å, could be indexed to the (111) planes of cubic diamond. The single line is drawn to indicate that the fringes do not extend from one hemisphere to the other, implying that these two crystalline parts developed independently.

1D fringes with a *d*-spacing of about 2.1 Å were observed in many nanoparticles. Although this can be indexed to (111) of the cubic diamond, it could also result from other phases. For example, another allotrope of carbon is hexagonal diamond or lonsdaleite. The *d*-spacings of the (100) and (002) planes of lonsdaleite are 2.18 and 2.06 Å, respectively. Both values are close to 2.10 Å. The 2.1 Å *d*-spacing can also fit to *d*-spacings of many other compounds, although the syntheses and EDX were very carefully performed to make sure no contaminants existed in the samples. Consequently, using 1D lattice images to identify a crystalline phase is more or less hypothetical. 2D images can offer three independent parameters in the image of each particle; two *d*-spacings and one corresponding interplane angle. It is therefore much more conclusive to use 2D HRTEM images to identify crystalline phases.

Figure 3c shows a 2D HRTEM image of a nanocrystallite. The measured *d*-spacings are *d*_B_ = 2.06 and *d*_C_ = 1.79 Å, which can be indexed to the (111) and (200) planes of a cubic diamond structure. The measured angle between these two groups of fringes is 55°, which also matches the theoretical interplanar angle of the planes in question. Figure 3d shows an HRTEM image of another cubic diamond particle. The two marked fringes have the same *d*-spacing of 2.05 Å, indexed to the (111) and (11̅1) planes of cubic diamonds with a corresponding interplane angle of 70°.

**Figure 4:**
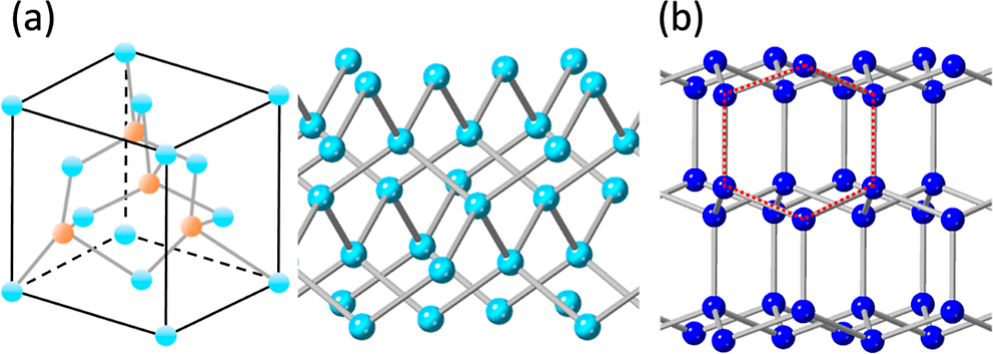
(a) Structural model of a cubic diamond. The orange spheres in the unit cell represent partial atomic vacancies. (b) Structural model of hexagonal diamond (lonsdaleite). The dashed red lines show a cyclohexane ring, which can be generated during the face-to-face connection of the graphene-like flakes.

According to the ideal structure of cubic diamond (Figure 4a), the (200) diffraction peak is systematic absent. One explanation for the observation of the (200) peak is that the crystal may contain many point defects, leading to a reduction of symmetry. A proposed structural model involves partially removing 4 carbon atoms at the inner sites, colored orange in the unit cell of cubic diamond (Figure 4a), changing the symmetry from *Fd*3*m* to *Fm*3*m*, the so-called n-diamond structure.^37,38^ In our opinion, the formation of these point defects (atomic vacancies) is due to the crystal growth process of multiple nucleation, aggregation, and recrystallization, as discussed above. The resolution of the electron microscope that we used was not high enough to directly observe point defects. However, point defects, either vacancies or interstitial excess atoms, would generate a local lattice distortion. Diffraction contrast at these distorted sites normally gives dark spots. Some examples of detecting excess oxygen atoms in metal oxides were demonstrated in previous reports.^39,40^ It is noted that the HRTEM contrast patterns of the nanodiamond particles (Figure 3c,d) are not even but contain some dark spots (Figure 3d, inset), which can be regarded as the sites of carbon vacancies.

**Figure 5:**
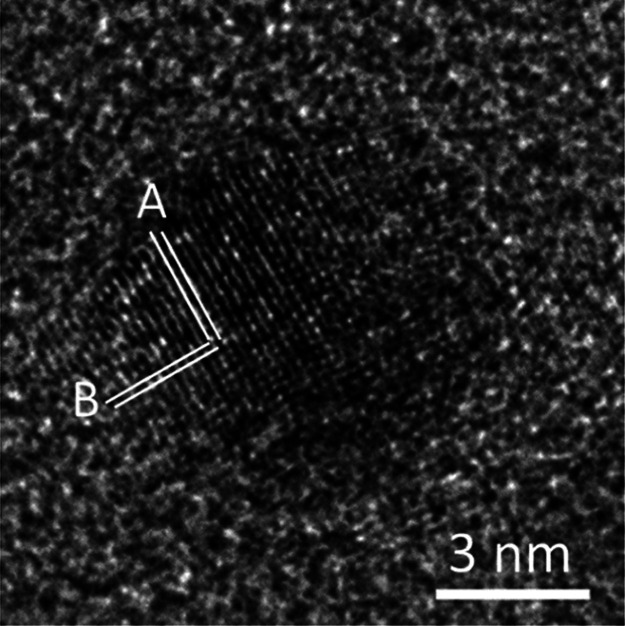
HRTEM image of a crystallite of a hexagonal diamond. The *d*-spacings of the fringes are measured to be *A* = 2.18 Å and *B* = 2.06 Å. The corresponding interplane angle is 90°.

There are a few 2D HRTEM images of nanocrystallites with *d*-spacings that do not match any atomic planes in cubic diamond. However, these *d*-spacings can be indexed to a hexagonal diamond (lonsdaleite, see Figure 4b). For example, the crystallite in Figure 5 shows two *d*-spacings, *d*_A_ = 2.18 and *d*_B_ = 2.06 Å. The corresponding interplanar angle is 90°. This pattern cannot be indexed to cubic diamond, but can be indexed to lonsdaleite with *A* = (100) and *B* = (002). The experimental values of the *d*-spacings match the theoretical values very well. Németh et al. believed that lonsdaleite is faulted and twinned cubic diamond and does not exist as a discrete material.^41^ However, our work indicates that a hexagonal nanodiamond (lonsdaleite) can exist by itself.

**Figure 6:**
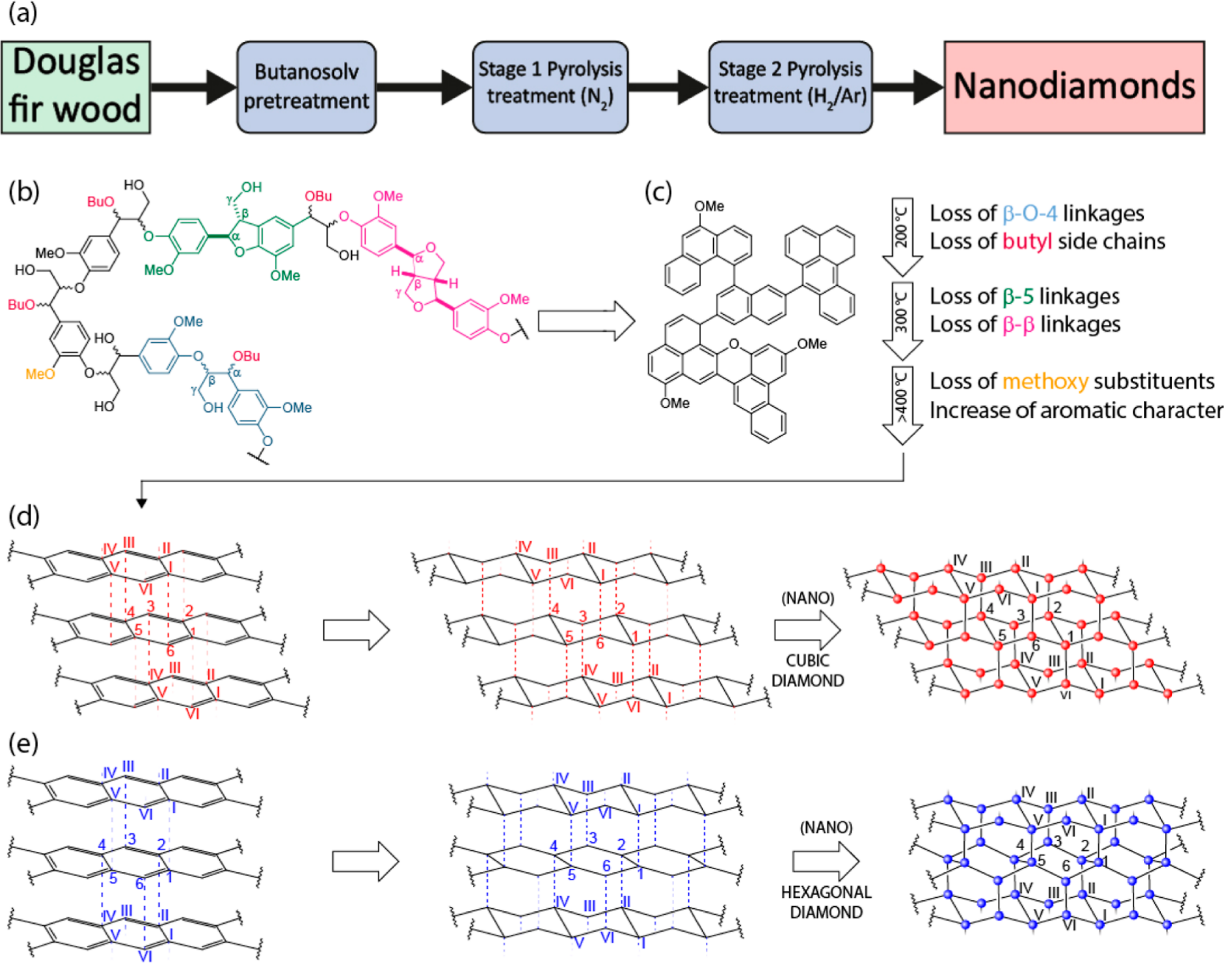
(a) Schematic representation of the experimental procedure used to prepare the nanodiamonds. See Figure S2 for an exploration of the impact of heating rate on lignin pyrolysis. (b) Representative structure of butanosolv lignin chain. (c) Representative structure of increasingly aromatic material resulting from the thermal decomposition of lignin. (d) Face-to-face interaction between two partially overlapped aromatic rings to form cubic diamond. (e) Full overlapped aromatic rings link together to form a hexagonal diamond.

Based on the experimental results in the present work and inspired by other reports, we considered the possible phase transformations that could be occurring on going from lignin to diamond. Lignin is an aromatic ring-containing polymer with a complex three-dimensional network structure. It consists of phenylpropanoid units connected by ether and carbon–carbon bonds. According to a large number of biosynthetic studies, lignin in plants is formed by the dehydrogenative polymerization of differing amounts of the precursors *p*-coumaryl alcohol **1**, coniferyl alcohol **2,** and sinapyl alcohol **3** (Figure S4) with the relative ratios of these being dependent on the biomass type.^42^ In softwoods, such as Douglas Fir used here, the major biosynthetic precursor is coniferyl alcohol **2**. During the lignification process, these small phenylpropanoid units link together through the formation of C–O–C and C–C bonds to give the important linkages in lignin, including the β-O-4, β-5, β–β, 5-5, 4-O-5, and β-1 bonds (Figure 6b). In this study, as the lignin used was a butanosolv lignin, the β-O-4 units have also been modified during the pretreatment to incorporate butanol at the α-position. It seems likely that, during pyrolysis, the structural units in lignin that contain aromatic rings have the potential to link further to give 2D defect graphene-like flakes (Figure 6c). FTIR and NMR analyses of samples prepared at low pyrolysis temperatures in this work support the view that, for example, the β-O-4 unit is no longer present at a (partial) pyrolysis temperature of 200, and at 300 °C, no NMR-detectable lignin units remain, although some methoxy groups are still present when the (partial) pyrolysis temperature is 300 °C. An increase in the FTIR signals corresponding to aromatic C=C bonds (relative to IR signals corresponding to, e.g., ether C–O bonds) compared to the starting lignin is consistent with increased aromatic ring formation during this low temperature phase. In these defect graphene-like flakes, the relevant orbitals on the carbon atoms are sp^2^-hybridized, and each flake is flat.

The aromatic rings, however, can also stack together by using face-to-face (π–π) intermolecular interactions, forming graphite-like structures. As the sample is heated to higher temperatures, dearomatization and radical–radical coupling may lead to new C–C bonds that link the flakes together. As shown in Figure 6d, if two partially overlapped aromatic rings (labeled 1 to 6 in one flake and I to VI in the neighboring flake, respectively) formed new bonds between, for example, C(1)–C(VI) and C(3)–C(IV), then the formation of armchair conformers of cyclohexane rings could occur. Extending this concept in three dimensions gives a crystal with a cubic diamond structure.

If two aromatic rings are fully overlapped and join together by forming, for example, C(1)–C(I), C(3)–C(III), and C(5)–C(V), as shown in Figure 6e, the final crystal that develops from such a nucleus has the lonsdaleite hexagonal diamond structure.

At high temperature, both processes presented in Figure 6d,e are possible, with the likelihood of partial overlap of aromatic rings being higher. Consequently, the formation of a cubic diamond dominates in the phase transformation. It is believed that defects in the graphite-like flakes play an important role in the phase transformation from lignin to diamond. In graphite, π electrons delocalize across whole graphene sheets to stabilize the flat morphology. Phase transformation from low density (2.3 g/cm^3^) graphite to high density (3.5 g/cm^3^) diamond requires a change from aromatic rings in the graphite sheets to armchair-shaped cyclohexane rings. The larger the dimensions of the graphite crystals, the more energy that is needed to perturb the C–C bonds. Therefore, the defect graphene-like nanoflakes can deform more easily from flat aromatic rings to armchair-shaped cyclohexane rings. Consequently, the phase transformation from lignin to diamond requires milder conditions than those from graphite.

## Conclusions

In summary, we communicate our recent successful fabrication of nanodiamonds embedded in an amorphous carbon substrate via the pyrolysis of lignin. The synthetic method is convenient and occurs at relatively low temperature without using high pressure. The only raw material is lignin extracted from biomass and the product contains only allotropes of carbon. The nanodiamonds are stable and potentially collectable. The proposed formation mechanism of diamonds from lignin based on the connection of defect graphene-like flakes in two different stacking modes implies that both cubic and hexagonal nanodiamonds could form. Mixed phases could also form if stacking faults occur, although we have not yet observed this structure. This work also implies that diamond crystals can form via packing of graphene-like nanoplates and breaking C=C bonds in aromatic rings and forming interplate C–C bonds along the packing direction. It can be reasonably hypothesized that nanoscale graphene and some aromatic ring-containing flat organic compounds may also serve as precursors for such phase transformations. The present work highlights an avenue for development of nonmetal nanoparticle-containing carbonaceous materials. Future work will require a detailed study of the breadth of this methodology, including attempts to assess the yield of nanodiamonds as a function of the type of starting lignin. For example, an expanded study on the use of commercially available lignins will be carried out. In addition, further investigation is required to determine an application for the nanodiamonds prepared in this work using conditions milder than those for commercially controlled detonation processes.

## Methods

### Materials

*n*-Butanol was obtained from Rathburn Chemicals Ltd., UK. Douglas fir sawdust was obtained from Hot Smoked (Useful Stuff Ltd.), UK. All materials were used as received.

### Butanosolv Pretreatment

Douglas fir sawdust was suspended in *n*-butanol/4 M HCl (95:5, 10 mL/g). The mixture was heated to reflux for 6 h and then filtered under vacuum, washing the pulp with acetone (2 × 5 mL/g), and then the filtrate was concentrated in vacuo, dissolved in the minimum volume of 9:1 acetone/water, and precipitated into water (10 v/v equiv). Anhydrous sodium sulfate was added as a flocculant to aid recovery of the lignin. The precipitate was collected by filtration, washed with excess water, and then dried in vacuo at 60 °C for 24 h. The dried residue was dissolved in the minimum volume of 9:1 acetone/methanol and precipitated by dropwise addition into 1:1 hexane/diethyl ether (10 v/v equiv). The precipitate was collected by filtration and dried in vacuo at 60 °C for 24 h to afford butanosolv lignin.

### Pyrolysis of Lignin

Butanosolv lignin was ground into a fine powder using a mortar and pestle and loaded into an alumina crucible. The lignin was heated in a tube furnace to the desired temperature at a heating rate of 10 °C min^–1^ in a N_2_ atmosphere (0.5 mL min^–1^) and then held at the desired temperature for 1 h before cooling down to room temperature. As a comparison, different heating rates of 5 and 20 °C min^–1^ were also applied. The obtained sample was then ground to a fine powder using a mortar and pestle and then resubmitted into the tube furnace. The sample was heated to 1050 °C for 1 h at a heating rate of 10 °C min^–1^ in a 5% H_2_/Ar mixed gas atmosphere (0.5 mL min^–1^). A final product of carbon was collected upon cooling to room temperature.

### Characterization Methods

FTIR spectra were collected on a Shimadzu IRAffinity 1S IR Spectrometer. 2D HSQC NMR spectra were recorded on a Bruker AV-III-HD 700 spectrometer fitted with CryProbe Prodigy TCI. The structural analysis of the specimens was initially examined using powder XRD on a STOE diffractometer in Debye–Scherrer (capillary) mode at room temperature using Mo Kα1 (λ = 0.709 Å) radiation. SEM images were recorded using a JEOL JSM-IT800 microscope operated from 5 to 15 kV. Samples were not coated with a conductive coating layer, so the original surface of the samples was observed. EDX was performed on the same electron microscope operated at a current of 15 kV. EDX samples were deposited on silica substrates, instead of carbon tapes, without a gold coating to avoid detecting external carbon and gold. TEM and HRTEM images were obtained using the FEI Titan Themis 200 S/TEM microscope (in TEM mode) operated at 200 kV. Powder samples for TEM/HRTEM were dispersed in acetone and deposited onto TEM specimen grids coated with a holey carbon film before being put into the sample chamber. TGA data were obtained using a Stanton Redcroft STA-780 simultaneous TG-DTA. Samples were dried under a vacuum at 50 °C for 24 h prior to TGA. Approximately 8 mg of sample was weighed into the crucible. Measurements were performed under a nitrogen flow (22 mL/min) at a heating rate of 10 °C/min.

## Supporting Information Available

2D HSQC NMR, SEM, EDX, powder XRD, TGA and DTG of lignin and pyrolyzed lignin samples and chemical structures of phenylpropanoid units in lignin (PDF)
