# Supplementary material for: Formation of Nanodiamonds during Pyrolysis of Butanosolv Lignin
Source: ACS Nano. 2024 Aug 23;18(36):24803–11. doi: 10.1021/acsnano.4c02950 (PMC11394345; doi:10.1021/acsnano.4c02950)
Supplement: Supplementary file 1 — nn4c02950_si_001.pdf [file nn4c02950_si_001.pdf]

Supporting Information

Formation of Nanodiamonds During Pyrolysis of Butanosolv Lignin

Yi Feng,†[a] Daniel J. Davidson,†[a],[b] Weihao Sun,[a] Valentina Milani, [a] Grant W. Howieson, [a] Nicholas J. Westwood,*[a],[b] and Wuzong Zhou*[a]

† These authors contributed equally.

* Corresponding Authors.

[a] EaStChem, School of Chemistry, University of St Andrews, St Andrews, Fife, KY16 9ST, UK.

[b] Biomedical Sciences Research Complex, University of St Andrews, North Haugh, St Andrews, Fife, KY16 9ST, UK.

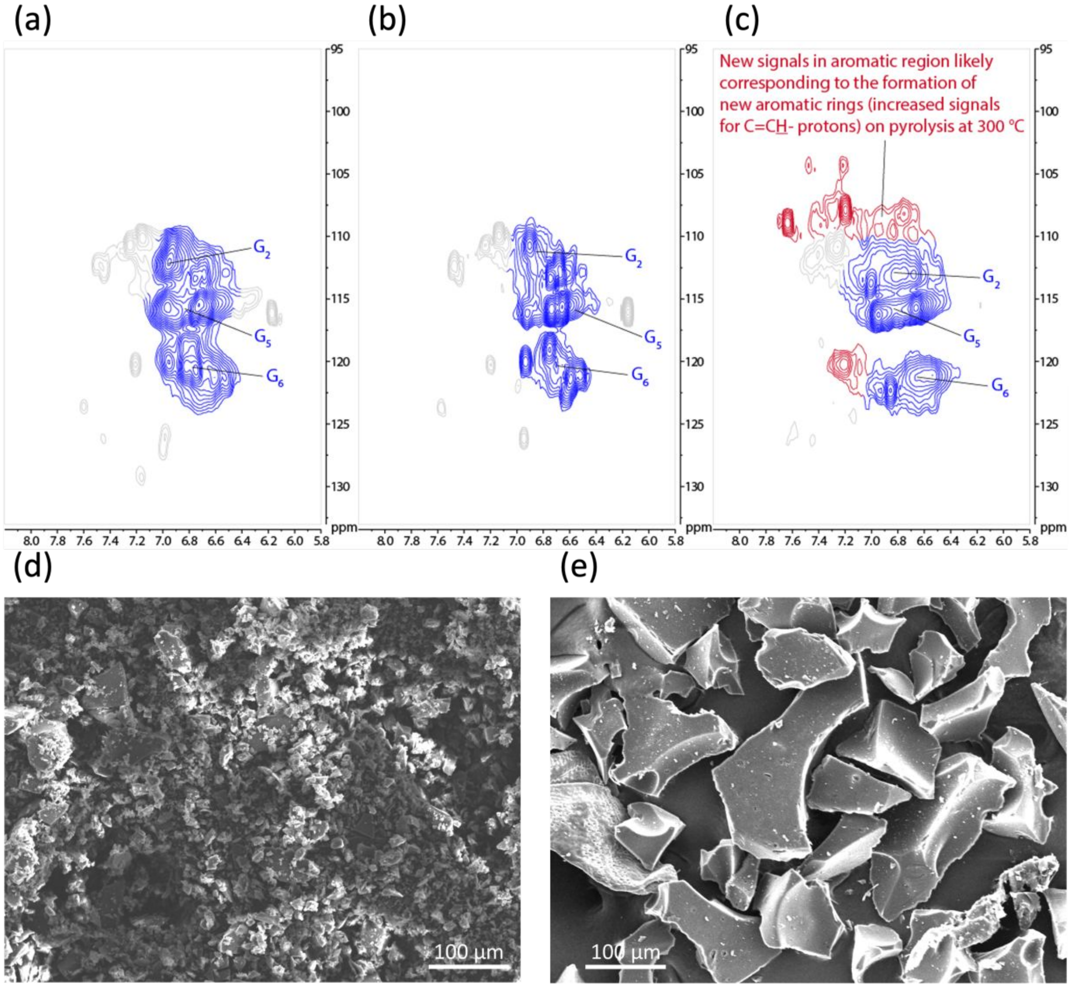

Figure S1. HSQC NMR (700 MHz, DMSO-d$_6$) spectra of the aromatic region of (a) butanosolv lignin before pyrolysis; (b) DMSO-soluble component of residue after pyrolysis at 200 °C; (c) DMSO-soluble component of residue after pyrolysis at 300 °C. The red signals in (c) likely correspond to the appearance of additional aromatic rings in the sample (increased signals for C=CH protons) upon heating to 300°C as proposed in Figure 6. The formation of additional aromatic rings is consistent with the formation of the proposed highly defected graphene-like flakes. SEM images of (d) starting butanosolv lignin showing its globular structure and (e) final carbon specimen showing the shard-like structure of the final sample. The globular structure of the starting lignin is consistent with, for example, the previously reported structure of an ethanosolv lignin.[S1]

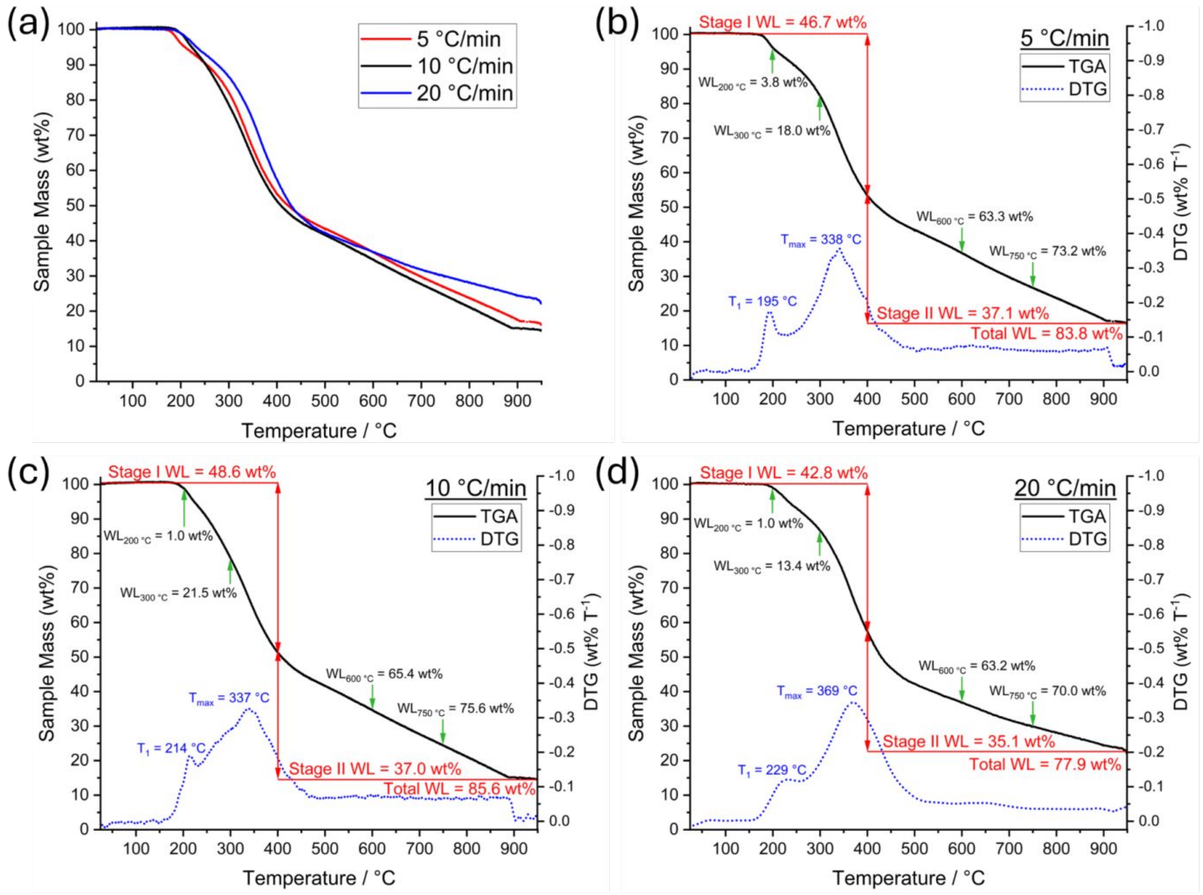

Figure S2. Experiments to assess the effect of different heating rates on the pyrolysis of Douglas Fir butanosolv lignin. (a) TGA curves of starting butanosolv lignin under N_2 atmosphere at various heating rates. TGA (solid black line) and DTG (dashed blue line) curves of starting butanosolv lignin under N_2 atmosphere at (b) 5°C min^{-1}, (c) 10°C min^{-1} and (d) 20°C min^{-1}. Pyrolysis was complete by 950°C with a heating rate of both 5°C min^{-1} and 10°C min^{-1}. Pyrolysis was not complete with a heating rate of 20°C min^{-1} as mass loss had not stopped by 950°C. Heating rate of 10°C min^{-1} was chosen for sample preparation as it was more practical to prepare multiple samples than 5°C min^{-1}.

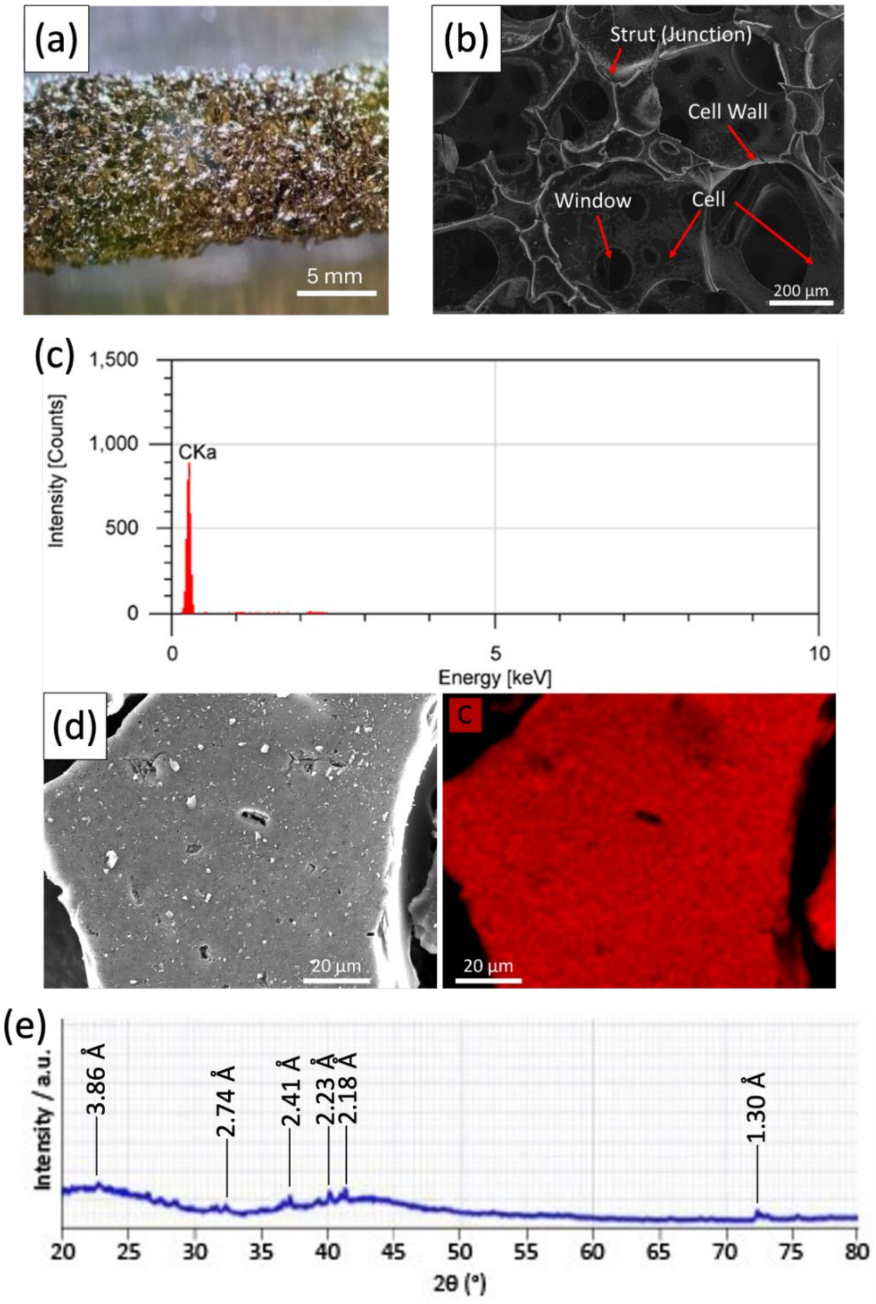

Figure S3. Initial characterization of the final carbon specimen after (i) pyrolysis in N_2 at 1000°C, followed by (ii) a second thermal treatment in 5% H_2/Ar at 1050°C, both at ambient pressure. (a) A digital photograph of the carbon specimen. (b) SEM image showing the porous structure of the material. (c) EDX spectrum of a focused region of the final sample. (d) SEM image and corresponding EDX mapping of a selected region of the final sample showing the predominant presence of carbon (red) across the relatively large analysed area (although the existence of hydrogen cannot be ruled out). (e) Powder XRD pattern of the sample. Some corresponding *d*-spacings to the visible peaks are indicated.

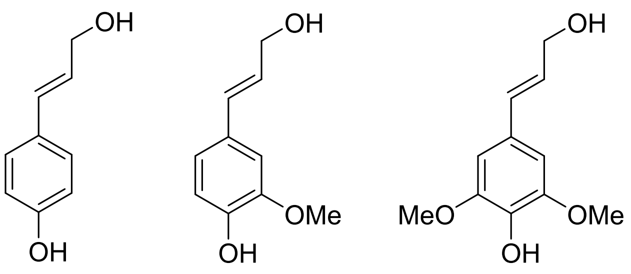

1 **2** **3**

Figure S4. Chemical structures of lignin biosynthetic precursors. **1** = *p*-coumaryl alcohol, **2** = coniferyl alcohol and **3** = sinapyl alcohol.

Reference

S1. Farid, M. A. A.; Zheng, A. L. T.; Tsubota, T.; Andou, Y. Catalytic Graphitization of Biomass-Derived Ethanosolv Lignin Using Fe, Co, Ni, and Zn: Microstructural and Chemical Characterization. *J. Anal. Appl. Pyrolysis* **2023**, *173*, 106064.
